# Robot-Assisted Radical Cystectomy with Ureterocutaneostomy: A Potentially Optimal Solution for Octogenarian and Frail Patients with Bladder Cancer

**DOI:** 10.3390/jcm14144898

**Published:** 2025-07-10

**Authors:** Angelo Porreca, Filippo Marino, Davide De Marchi, Alessandro Crestani, Daniele D’Agostino, Paolo Corsi, Francesca Simonetti, Susy Dal Bello, Gian Maria Busetto, Francesco Claps, Aldo Massimo Bocciardi, Eugenio Brunocilla, Antonio Celia, Alessandro Antonelli, Andrea Gallina, Riccardo Schiavina, Andrea Minervini, Giuseppe Carrieri, Antonio Amodeo, Luca Di Gianfrancesco

**Affiliations:** 1 Department of Biomedical Sciences, Humanitas University, 20072 Milan, Italy; angelo.porreca@hunimed.eu; 2 Department of Urology, Humanitas Gavazzeni, 24125 Bergamo, Italy; davide.demarchi@gavazzeni.it (D.D.M.); francesca.simonetti@gavazzeni.it (F.S.); luca.digianfrancesco@gavazzeni.it (L.D.G.); 3 Department of Urology, Ospedale Santa Maria Della Misericordia di Udine, 33100 Udine, Italy; alessandro.crest@gmail.com; 4 Department of Urology, Villa Salus Clinic, 30174 Mestre, Italy; dott.dagostino@gmail.com; 5 Department of Urology, Veneto Institute of Oncology (IOV)—IRCCS, Headquarter of Castelfranco Veneto, 31033 Padua, Italy; paolo.corsi@iov.veneto.it (P.C.); susy.dalbello@iov.veneto.it (S.D.B.); antonio.amodeo@iov.veneto.it (A.A.); 6 Department of Urology, University of Foggia, 71122 Foggia, Italy; gianmaria.busetto@unifg.it (G.M.B.); giuseppe.carrieri@unifg.it (G.C.); 7 Department of Urology, University or Trieste, Cattinara Hospital–ASUGI, 34149 Trieste, Italy; claps.francesco@gmail.com; 8 Department of Urology, ASST Grande Ospedale Metropolitano Niguarda, 20162 Milan, Italy; aldomassimo.bocciardi@ospedaleniguarda.it; 9 Division of Urology, IRCCS Azienda Ospedaliero Universitaria di Bologna, 40138 Bologna, Italy; eugenio.brunocilla@unibo.it (E.B.); riccardo.schiavina3@unibo.it (R.S.); 10 Department of Urology, San Bassiano Hospital, 36061 Bassano del Grappa, Italy; antonio.celia@aulss7.veneto.it; 11 Department of Urology, University of Verona, Azienda Ospedaliera Universitaria Integrata, 37126 Verona, Italy; alessandro.antonelli@univr.it; 12 Department of Urology, Ospedale Regionale di Lugano, Civico USI–Università della Svizzera Italiana, 6900 Lugano, Switzerland; andrea.gallina@eoc.ch; 13 Department of Experimental and Clinical Medicine, University of Florence, 50121 Florence, Italy; andrea.minervini@unifi.it

**Keywords:** bladder cancer, complications, elderly, morbidity, robotic cystectomy, ureterocutaneostomy

## Abstract

**Background/Objectives:** Robot-assisted radical cystectomy (RARC) has become the primary approach for treating bladder cancer, replacing the traditional open procedure. The robotic approach, when combined with ureterocutaneostomy (UCS), offers significant advantages for octogenarians, who are at increased risk for perioperative complications. **Methods:** This observational, prospective, multicenter analysis is based on data from the Italian Radical Cystectomy Registry (RIC), collected from January 2017 to June 2020 across 28 major urological centers in Italy. We analyzed consecutive male and female patients undergoing radical cystectomy (RC) and urinary diversion via the open, laparoscopic, or robot-assisted technique. Inclusion criteria: patients aged 80 years or older, with a WHO Performance Status (PS) of 2–3, an American Society of Anesthesiologist score ≥3, a Charlson Comorbidity Index (CCI) ≥ 4, and a glomerular filtration rate (GFR) <60 mL/min. **Results:** A total of 128 consecutive patients were included: 41 underwent RARC with UCS (Group 1), 65 open RC (ORC) with UCS (Group 2), and 22 laparoscopic RC (LRC) with UCS (Group 3). The cystectomy operative time was longer in robotic surgeries, while the lymph node dissection time was shorter. RARC with UCS showed statistically significant advantages in terms of lower median estimated blood loss (EBL), transfusion rate, and length of hospital stay (LOS) compared to open and laparoscopic procedures. Intra- and postoperative complications were also lower in the RARC groups. **Conclusions:** Robotic cystectomy in high-volume referral centers (≥20 cystectomies per year) provides the best outcome for fragile patients. Beyond addressing the baseline pathology, RARC with UCS may represent a leading option, offering oncological control while reducing complications in this vulnerable age group.

## 1. Introduction

Bladder cancer (BCa) is a prevalent and aggressive malignancy within the field of urology, particularly affecting older adults [1]. For patients with muscle-invasive bladder cancer (MIBC) or high-risk recurrent non-muscle-invasive bladder cancer (NMIBC), radical cystectomy (RC) remains the standard therapeutic approach [2]. However, RC is associated with considerable perioperative morbidity and mortality, especially among elderly and frail patients [3]. Given the rise in life expectancy, it is increasingly important to refine surgical strategies to improve safety and outcomes in this vulnerable cohort. Although minimally invasive surgical options have become more common, there is still no clear consensus on the best surgical approach for frail elderly patients in terms of operative safety, postoperative recovery, and long-term feasibility.

Historically, open radical cystectomy (ORC) has served as the conventional method for treating MIBC. While effective in terms of cancer control, it is linked with significant perioperative risks, such as high intraoperative blood loss, extended hospital stays, and a high incidence of complications. These issues are particularly critical for octogenarians and patients with multiple comorbidities. To address these challenges, minimally invasive techniques, such as laparoscopic radical cystectomy (LRC) and robot-assisted radical cystectomy (RARC), have gained widespread adoption in the field of urological oncology.

RARC has become a recognized alternative to open surgery, offering improvements in surgical accuracy and oncological outcomes, along with lower rates of perioperative complications. Notably, RARC is associated with benefits such as shorter hospital stays, a reduced need for blood transfusions, and quicker recovery times [4,5,6,7]. These advantages are especially pertinent in elderly and frail patients, who are more susceptible to the risks posed by traditional open approaches. Furthermore, when combined with ureterocutaneostomy (UCS), RARC presents a potential ideal solution for managing BCa in the elderly, possibly redefining the standard of care for this population [8].

This prospective observational study aims to evaluate and compare the clinical outcomes of three different surgical approaches to radical cystectomy—RARC, LRC, and ORC—when combined with ureterocutaneostomy (UCS), specifically in elderly and frail patients with BCa. The primary objective is to address the perioperative safety and feasibility of these techniques in this vulnerable population. Key endpoints include the rate of perioperative complications, 30-day hospital readmission, and 90-day postoperative mortality. By systematically analyzing these outcomes, the study aims to clarify whether robotic and minimally invasive approaches can offer superior safety profiles and faster recovery compared to traditional open surgery. Furthermore, the study intends to confirm the feasibility of these advanced techniques even in frail and elderly patients, who are often considered unsuitable for complex surgical interventions.

## 2. Materials and Methods

### 2.1. Patient Population and Study Design

This study represents a sub-analysis focusing on frail patients undergoing RC, extracted from the Italian Radical Cystectomy Registry (RIC). The RIC is a prospective, observational, multicenter cohort study conducted across 28 major urological centers in Italy from January 2017 to June 2020 [9,10].

For this sub-analysis, the inclusion criteria were strictly defined to select frail patients. Eligible participants included those aged 80 years or older, with World Health Organization (WHO) Performance Status (PS) of 2–3, an American Society of Anesthesiologist (ASA) score of ≥3, a Charlson Comorbidity Index (CCI) ≥4, and a glomerular filtration rate (GFR) of <60 mL/min. These thresholds were defined a priori by the study group, based on clinical experience and the prior literature describing frailty and surgical risk in elderly urological patients [11]. The most frequent comorbidities contributing to the CCI score were hypertension (56.3%), diabetes mellitus (32.0%), and heart failure (14.1%), confirming the clinical complexity of the study population.

Exclusion criteria included patients younger than 80 years, individuals not meeting the defined frailty criteria, those with evidence of metastatic disease, or those unable to provide informed consent. Patients undergoing salvage cystectomy were excluded from the analysis.

All patients underwent a preoperative thoraco-abdominal CT scan to exclude metastatic disease. The selected patients were categorized into three groups based on the surgical approach employed: RARC with UCS (Group 1), ORC with UCS (Group 2), and LRC with UCS (Group 3). Group allocation was determined at the discretion of the operating surgeon and guided by several factors including the anatomical and clinical characteristics of each patient and the availability of specific surgical technologies and resources at the respective centers. To ensure consistency and reduce variability, the surgeries were performed by surgeons with expertise in this field at the respective centers.

All participating centers were high-volume referral institutions, each performing over 20 radical cystectomies annually. Surgical procedures were conducted by experienced urologic surgeons with consolidated expertise in both open and minimally invasive pelvic surgery, ensuring technical consistency across the cohort. All procedures followed standardized protocols defined within the framework of the Italian Radical Cystectomy Registry (RIC). Enhanced Recovery After Surgery (ERAS) protocols were uniformly implemented across all centers to optimize perioperative outcomes

Postoperatively, patients were followed up with visits at 30 days, 3 months, and subsequently every 6 months. Follow-up assessments included imaging studies such as CT scans or ultrasound. However, a standardized protocol for follow-up timing was not formally established, allowing some variability in practice.

Data collected included:

• Preoperative data: age, body mass index (BMI), WHO PS, ASA score, CCI, and preoperative creatinine value.
• Perioperative data: surgical time for RC and lymph node dissection, estimated blood loss (EBL), transfusion rate, and intraoperative complications.
• Postoperative data: postoperative complications classified according to a Clavien–Dindo (CD) classification grade ≥ 4, length of hospital stay (LOS), 30-day readmission rate, and 90-day mortality rate.

Data collection adhered to the World Medical Association Declaration of Helsinki, with all patients providing informed consent. The study was approved by the Ethical Committee of the University of Padova (approval number: 0042389, 25 June 2020) and registered on ClinicalTrials.gov under reference number NCT04228198.

**Figure 1:**
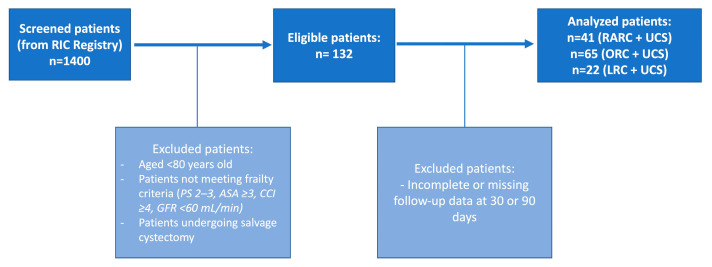
Flowchart illustrating the selection of patients included in the study. From the original cohort of the Italian Radical Cystectomy Registry (RIC) (~1400 patients), only frail patients meeting strict clinical criteria (age ≥ 80, PS 2–3, ASA ≥ 3, CCI ≥ 4, GFR < 60 mL/min) were included. The final population consisted of 128 patients, stratified by surgical technique into RARC (n = 41), ORC (n = 65), and LRC (n = 22).

A flowchart summarizing the selection process of the study population is provided in Figure 1.

### 2.2. Surgical Technique

Urinary diversion was performed via ureterocutaneostomy (UCS), tailored to the surgical approach and intraoperative anatomical conditions. In all three approaches—RARC, LRC, and ORC—the ureters were dissected distally and divided near the bladder. The UCS was then constructed either as a bilateral cutaneous ureterostomy, with both ureters brought separately to the skin and matured individually as stoma nipples, or as a unilateral Wallace-type stoma, in which the ureters were spatulated and anastomosed side-by-side to create a single plate, then exteriorized through a single abdominal site.

In RARC, the left ureter was routinely transposed beneath the sigmoid mesocolon to reach the right side, allowing the creation of a single stoma. The decision between the bilateral and unilateral configurations was based on ureteral length, anatomy, and intraoperative feasibility. In LRC, the diversion was performed extracorporeally through a small infraumbilical incision, whereas in ORC, it was executed through the midline laparotomy. In all cases, single-J stents were inserted over guidewires and fixed externally with non-absorbable sutures. Stents were managed on an outpatient basis and routinely replaced every 3–6 months.

### 2.3. Study Outcomes

The study assessed multiple clinically meaningful outcomes to compare the safety and efficacy of different surgical approaches.

Primary endpoints included (1) EBL, (2) LOS, (3) intraoperative complications, and (4) postoperative transfusion rate. These metrics were selected as direct indicators of perioperative morbidity and surgical performance.

Secondary endpoints encompassed (a) the 30-day hospital readmission rate, (b) 30- and 90-day postoperative complications (classified according to the Clavien–Dindo system), and (c) 90-day all-cause mortality.

The 30-day readmission rate was defined as any unplanned hospital admission occurring within 30 days of discharge, irrespective of cause. This measure was used as a standardized indicator of early postoperative safety and resource utilization, in line with the recent literature [12].

Ninety-day mortality was evaluated through Kaplan–Meier survival analysis. Time-to-event was calculated from the date of surgery to death or the last follow-up. Patients who were alive at the end of follow-up were censored. Survival curves were compared across surgical groups using the log-rank test.

### 2.4. Statistical Analysis and Reporting

Demographic, perioperative, and follow-up data were analyzed using descriptive statistics techniques. We analyzed the baseline characteristics to compare group 1 with the other groups regarding perioperative outcomes such as surgical time, EBL, need for transfusion, surgical complications, and LOS. Quantitative variables were expressed as median and the first and third quartile (q1–q3). The normality of continuous variables was assessed using the Shapiro–Wilk test. Since most variables did not follow a normal distribution, data were summarized as medians and interquartile ranges (q1–q3), and non-parametric tests were used for statistical comparisons.

Qualitative variables were presented as absolute and relative frequencies (percentages). Parametric and nonparametric variables were evaluated using ANOVA and Kruskal–Wallis, respectively. Statistical significance was defined as a two-sided *p*-value < 0.05. We conducted all analyses using the statistical software STATA/SE version 18 (StataCorp, College Station, TX, USA).

The study was reported in compliance with the Strengthening the Reporting of Observational Studies in Epidemiology (STROBE) guidelines (Supplementary Materials).

## 3. Results

A total of 128 consecutives patients were included in the analysis. Overall, 41 patients underwent RARC with UCS (Group 1), 65 underwent ORC with UCS (Group 2), and 22 underwent LRC with UCS (Group 3).

Table 1 summarizes the baseline characteristics of the three groups. No statistically significant differences were observed among the groups in terms of preoperative characteristics, including age, BMI, ASA score, CCI, and preoperative creatinine levels.

Table 2 outlines the perioperative and postoperative outcomes for the three groups.

The median cystectomy surgical time was significantly longer for robotic procedures (140 min) compared to open (110 min) and laparoscopic approaches (105 min). The median lymph node dissection time was statistically shorter in the RARC group (40 min) compared to the open (52 min) and laparoscopic groups (60 min). Furthermore, there was no significant difference in the time required for urinary diversion among the groups.

**Figure 2:**
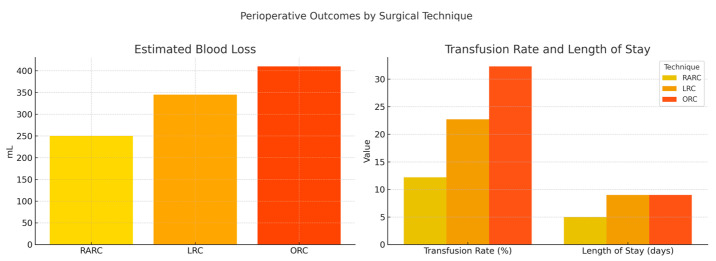
Estimated blood loss, transfusion rate, and hospital stay by surgical technique. Minimally invasive approaches (RARC and LRC) showed favorable trends compared to open surgery (ORC).

Both minimally invasive approaches, robotic and laparoscopic, showed a trend toward lower blood loss and transfusion requirements compared to the open technique. While the difference was statistically significant only between the robotic and open groups, the overall trend favors minimally invasive surgery. The median EBL in the robotic group was substantially lower (250 mL) compared to the open (410 mL) and laparoscopic (345 mL) groups, with the difference being statistically significant between the robotic and open groups. Correspondingly, the need for transfusion was significantly reduced in the robotic group, with only 12.2% of patients requiring transfusions, compared to 32.2% in the open group. These findings underscore the ability of robotic surgery to minimize intraoperative trauma and enhance perioperative safety. A graphical summary of estimated blood loss, transfusion rates, and hospital stay by surgical approach is shown in Figure 2.

Intraoperative complications were also less frequent in the robotic group, occurring in only 2.4% of cases, compared to 9.2% in the open group and 9.1% in the laparoscopic group. These complications included rectal injury and vascular injury, both of which were managed with intraoperative suturing. There was no intraoperative mortality reported in any of the groups. However, these differences were not statistically significant.

Severe postoperative complications (CD ≥ 4) were absent in the robotic group, while they occurred in 3.1% and 4.5% of patients in the open and laparoscopic groups, respectively. These complications consisted of urosepsis requiring monitoring in the intensive care unit. Again, these differences were not statistically significant.

**Figure 3:**
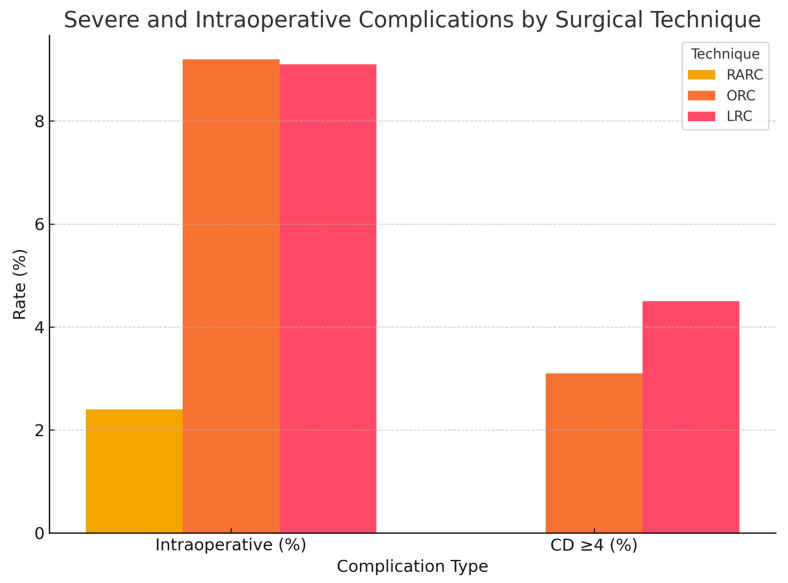
Comparison of intraoperative complication rates and severe postoperative complications (Clavien–Dindo grade ≥ 4) among patients undergoing RARC, ORC, and LRC. Lower complication rates were observed in the robotic group, though differences were not statistically significant.

A graphical summary of intraoperative and Clavien–Dindo ≥ 4 postoperative complications by surgical technique is presented in Figure 3.

Perhaps the most striking difference was observed in the length of hospital stay (LOS). Patients undergoing robotic surgery experienced a significantly shorter median LOS (5 days) compared to 9 days in both the open and laparoscopic groups.

Post hoc power analysis confirmed that the sample size provided sufficient statistical power to detect clinically meaningful differences for the main perioperative outcomes. Specifically, the estimated power was 92.8% for estimated blood loss (Cohen’s d = 0.688), 100% for length of stay (Cohen’s d = 1.579), 70.0% for transfusion rate (Cohen’s h = 0.495), and 33.4% for intraoperative complications (Cohen’s h = 0.305).

The 30-day readmission rate showed no statistically significant differences among the groups. However, the robotic group had the lowest rate (17.7%) compared to 20% in the open group and 18% in the laparoscopic group. Among the readmitted patients, the most frequent causes were urinary tract infections, wound-related complications, and stoma management issues. In the RARC group, seven patients were readmitted, primarily for urinary infections (n = 4), stoma-related problems such as bleeding or mild prolapse (n = 2), and wound infection (n = 1). In the ORC group, 13 patients experienced unplanned readmission, most commonly due to urinary infections (n = 5), wound complications (n = 4), cardiopulmonary events such as atrial fibrillation or pneumonia (n = 2), and stoma-related problems (n = 2). In the LRC group, four patients were readmitted, with causes including urinary tract infections (n = 2), minor stoma complications (n = 1), and wound dehiscence (n = 1).

The 90-day mortality rate was also lower in the robotic group (7.3%), compared to 10.7% in the open group and 9.1% in the laparoscopic group, although the differences were not statistically significant.

## 4. Discussion

Managing BCa in elderly and frail patients presents significant challenges due to the high morbidity and mortality associated with RC, particularly with open surgery. However, robotic surgery, with its minimally invasive approach, has substantially mitigated these risks. Our findings suggest that minimally invasive techniques—both robotic and laparoscopic—offer potential perioperative advantages over the open approach in frail and elderly patients, particularly in terms of reduced blood loss, shorter hospital stay, and lower transfusion rates. Although the difference between RARC and LRC was not statistically significant in most parameters, both approaches appear favorable when compared to traditional open surgery.

Laparoscopic surgery, once considered unsuitable for elderly patients due to concerns about its impact on cardiac and pulmonary function, is now supported by evidence demonstrating its benefit. Studies have shown that LRC offers advantages such as shorter hospital stays and fewer complications compared to open surgery [13,14]. Moreover, both LRC and RARC have been associated with a reduction in surgical site infections [15,16].

For older patients with MIBC, the decision to undergo RC often requires a careful balance between the potential loss of function and independence and the prospect of life extension. Factors influencing this decision include the presence of comorbidities, frailty, and psychological considerations. As patients age, their ability to tolerate aggressive treatment gradually diminishes, necessitating a personalized and nuanced approach to treatment planning [17].

Historically, RC has been underutilized in the treatment of MIBC, despite long-standing guidelines recommending it. A study using data from the Medicare database revealed that only 21% of MIBC patients underwent RC [18]. Similarly, a later analysis of the SEER database reported that only 6.9% of patients over the age of 80 received RC [18]. Notably, survival rates were significantly higher among patients who received treatment, raising concerns about the potential under-treatment of elderly patients.

It is increasingly recognized that chronological age alone should not be an absolute contraindication for RC. Evidence suggests that RC can be performed safely in older patients, though it is associated with higher rates of complications and mortality compared to younger cohorts [18,19].

The growing elderly population has generated interest in urinary diversions that carry lower postoperative risks, such as UCS [20,21]. First described in 1960, UCS has demonstrated the ability to reduce mortality and complication rates in older patients, with studies highlighting improved outcomes compared to other types of urinary diversions [22]. For example, De Nunzio et al. reported morbidity and mortality rates of 13% and 4%, respectively, in a series of octogenarians who underwent RC with UCS [23].

Adamczyk et al. [24] found that the type of urinary diversion influenced complication rates, whereas factors such as diabetes, renal insufficiency, and low albumin levels has less impact. Open surgical approaches were associated with a higher frequency of complications compared to minimally invasive techniques like LRC and RARC [24]. By eliminating the need for bowel anastomosis, UCS reduces the risk of postoperative ileus (POI), a common and often debilitating complication in more complex urinary diversions. Studies have consistently shown a lower incidence of POI with UCS, underscoring its role as a simpler and safer option for elderly and frail patients [25,26].

Beyond surgical factors, dietary patterns and nutritional status may also influence perioperative and long-term cancer outcomes. There is growing evidence that plant-based foods, particularly berries, contain bioactive compounds such as flavonoids and anthocyanins that exert antioxidant and anti-inflammatory effects [27]. These compounds may contribute to reducing oxidative stress, modulating immune responses, and influencing tumorigenesis. While our study does not specifically evaluate the role of diet in bladder cancer outcomes, further research could explore whether nutritional interventions, including antioxidant-rich foods, could positively impact perioperative recovery and long-term health in frail patients undergoing major urological surgery.

However, UCS is associated with a significant risk of stoma stenosis, which can result in urinary infections and long-term quality-of-life (QoL) challenges [28]. Although technical advancements have been made, maintaining stoma patency often requires the use of a catheter, which increases the risk of infection. Despite these limitations, UCS offers several advantages over ileal conduits, including shorter operative times, reduced blood loss, fewer transfusions, and decreased need for intensive care unit stays [29]. Originally developed for pediatric patients, UCS has since been adapted for adults with ureteral obstructions [30]. While simple and effective, UCS is limited by its high rates of UTIs and stoma stenosis, which have restricted its widespread use. Nonetheless, these drawbacks are often outweighed by its benefits, particularly for frail patients or those with solitary kidneys who require supravesical diversion. Moreover, UCS has been successfully integrated into robotic and laparoscopic approaches, further enhancing its applicability and safety for vulnerable patient populations [11,31,32,33].

The choice of urinary diversions following RC is typically guided by patient preferences, overall health status, and cancer treatment requirements. Among the available options, ileal conduits remain the most utilized method due to their relative simplicity and suitability for patients with significant health issues [34]. Early complications can include infections and wound problems, while long-term issues may involve urolithiasis and stomal complications, such as hernias [35,36].

Although UCS eliminates the need for bowel diversion, it is generally less continent than ileal conduits, often resulting in skin irritation caused by urine leakage. Furthermore, renal function tends to decline more rapidly in patients with UCS, making it a more suitable option for patients with limited life expectancy or multiple comorbidities [37]. For patients seeking a more continent alternative, catheterizable reservoirs provide a viable solution. These pouches improve continence and allow for more natural voiding, but they come with increased surgical complexity and potential risks, such as metabolic imbalances and pouchitis [38]. Urinary diversion techniques have undergone significant evolution over the past century, with the choice of method often depending on the surgeon’s expertise and the patient’s specific medical and lifestyle considerations. Determining the optimal approach for urinary tract reconstruction remains a complex challenge that requires a thorough discussion between the surgeon and the patient. Ongoing advancements in surgical techniques and continued research is expected to further enhance the safety and effectiveness associated with these procedures.

UCS has been described in several retrospective studies, demonstrating favorable outcomes regarding EBL and transfusion rates [25,39,40]. In our series, patients undergoing UCS showed relatively low EBL and lower transfusion requirements overall, which aligns with previous findings. Notably, the robotic UCS group exhibited longer operative times compared to the open and laparoscopic groups, a difference likely attributable to the learning curve associated with the adoption of robotic cystectomy. However, the robotic approach demonstrated a statistically significant reduction in EBL compared to the open approach, while it was lower but not statistically significant compared to laparoscopy. Additionally, the robotic approach showed a statistically significant reduction in transfusion rates compared to open surgery, further highlighting its potential benefits once surgical teams gain experience.

LOS for UCS has been reported to be relatively short in previous studies [25,39,41]. In our analysis, the robotic UCS group demonstrated a shorter median LOS compared to both the open and laparoscopic approaches, suggesting a potential advantage of the robotic technique.

Postoperative intensive care unit (ICU) requirements for UCS have been reported to be relatively low in the literature [25,36]. Additionally, the duration of abdominal drainage in UCS procedures has been described as relatively short (3.7 days), highlighting the potential favorable impact on patient recovery [25].

Complication rates further highlight the advantages of UCS, particularly with the robotic approach. Clavien–Dindo (CD) I–II complication rates for UCS patients have ranged from 17.7% [41] to 57.1% [25]. Severe complications (CD > 2) are reported at rates of 7.1% to 27.3% [25,41]. In our experience, the robotic UCS group showed relatively lower complication rates (2.4%) compared to the open (9.2%) and laparoscopic (9.1%) approaches, although this difference did not reach statistical significance.

QoL assessments offer additional insights. In the study by Longo et al. [25], the Bladder Cancer Index (BCI) was used to evaluate QoL, and the results demonstrated that patients undergoing UCS achieved QoL outcomes comparable to those observed in patients receiving ileal conduits. This finding is particularly significant for elderly patients with relevant comorbidities, as UCS is often perceived as inferior due to the need for periodic ureteral stent changes and the use of a dual urine collection system.

### 4.1. Limitations

Limitations of this study include its observational and multicenter design; there is potential heterogeneity in surgical practices and postoperative care across the participating centers. This variability may have influenced the observed outcomes, making it difficult to generalize the conclusions to all clinical settings. Another limitation is the relatively small sample size, particularly concerning the group undergoing laparoscopic radical cystectomy (LRC), which may have limited the statistical power of the analyses and the ability to detect significant differences between groups. Additionally, detailed long-term follow-up data were not included, limiting the assessment of long-term oncological and functional outcomes, such as disease-free survival and postoperative quality of life. Another limitation is the absence of systematic data regarding the preoperative use of anticoagulant or antiplatelet therapy, which may have influenced bleeding risk and transfusion rates in this elderly population.

The study did not include a pre-specified primary endpoint or an a priori sample size calculation, which limits formal hypothesis testing. Although a post hoc power analysis demonstrated sufficient power for key outcomes such as blood loss and hospital stay, the study was underpowered for rare events like intraoperative complications.

Finally, the lack of randomization in the study introduces potential selection bias, as more frail patients may have been directed toward robotic treatment (RARC) compared to traditional techniques, potentially skewing the results in favor of RARC. These limitations highlight the need for further randomized studies with long-term follow-up to confirm the benefits of RARC with UCS in frail elderly patients with bladder cancer.

Nevertheless, our study provides valuable insights into the national landscape of urinary diversion techniques. UCS represents a life-saving procedure that offers optimal oncologic treatment for BCa, without the significant morbidity and mortality risks associated with intestinal diversions in elderly and frail populations. By reducing the morbidity associated with RC through simpler urinary diversion techniques, we anticipate that more patients will benefit from this standard and optimal treatment for MIBC.

### 4.2. Perspectives

#### 4.2.1. Benefits of Robotic Cystectomy

1. Minimally Invasive Approach: Robotic surgery enables precise procedures through smaller incisions, minimizing surgical impact and facilitating quicker recovery.
2. Decreased Complications Rates: Research suggests fewer postoperative complications, including infections and bleeding, compared to traditional open cystectomy.
3. Shorter Recovery Times: Patients undergoing robotic cystectomy experience shorter hospital stays and a faster return to normal function.

#### 4.2.2. Ureterocutaneostomy: A Simplified Option

Ureterocutaneostomy, which involves the diversion of the ureters directly to the skin, is a comparatively straightforward procedure when compared with orthotopic reconstruction or a uretero–ileal conduit. This simplicity is particularly beneficial for elderly patients, who may not tolerate more complex surgeries.

1. Simplified Postoperative Care: Managing ureterocutaneostomy after surgery is less complex, lowering the likelihood of urinary complications.
2. Reduced Physiological Stress: By avoiding intricate reconstructions, there is less physiological strain on the patient, leading to improved postoperative outcomes, especially for those with significant comorbidities.

The combination of robotic cystectomy and ureterocutaneostomy presents a viable alternative for elderly patients with bladder neoplasia. The less invasive nature of robotic surgery and the simplicity of managing ureterocutaneostomy could significantly mitigate the risks associated with traditional procedures.

#### 4.2.3. Challenges and Considerations

While there are benefits, the implementation of robotic cystectomy with ureterocutaneostomy presents several challenges:

1. Cost: Robotic technology is expensive and may not be available in all healthcare facilities.
2. Learning Curve: Robotic surgery requires specialized training and involves a substantial learning curve.
3. Patient Selection: Thoughtful patient selection is essential to optimize the outcomes of this procedure, taking into account existing health conditions and other medical factors.

## 5. Conclusions

Robot-assisted radical cystectomy (RARC) with ureterocutaneostomy (UCS) represents a viable and safe surgical option for elderly and frail patients with bladder cancer. In our multicenter cohort, RARC was associated with significantly lower estimated blood loss, reduced transfusion rates, and shorter hospital stays compared to the open approach. No significant differences were observed among the three techniques in terms of major intraoperative complications, Clavien–Dindo ≥ 4 postoperative events, readmission rates, or 90-day mortality.

These findings suggest that RARC with UCS may offer tangible perioperative benefits in high-risk populations, particularly when performed in experienced, high-volume centers. Further studies are required to validate these results and define the long-term oncologic and functional outcomes of this approach.

## Figures and Tables

**Table 1:** Baseline characteristics.

Median (q1–q3)	RARC + UCS (41)	ORC + UCS (65)	LRC + UCS (22)	*p*-Value (RARC + UCS vs. ORC + UCS)	*p*-Value (RARC + UCS vs. LRC + UCS)
**Age (years)**	83.7 (80–88)	82.9 (81–85)	81.9 (80–85)	0.296	0.280
**BMI (kg/m^2^)**	26.4 (18–39)	24.2 (20–31)	24.5 (18–34)	0.246	0.560
**ASA score**	3 (3–4)	3 (3–4)	3 (3–4)	0.921	0.883
**CCI**	5 (4–7)	5 (4–7)	5 (4–7)	0.552	0.971
**Preoperative creatinine (mg/dL)**	1.4 (0.7–2.9)	1.5 (0.9–2.3)	1.3 (0.7–2.5)	0.681	0.689

RARC, robot-assisted radical cystectomy; ORC, open radical cystectomy; LRC, laparoscopic radical cystectomy; UCS, ureterocutaneostomy; BMI, Body Mass Index; ASA, American Society of Anesthesiologists; CCI, Charlson Comorbidity Index.

**Table 2:** Perioperative and postoperative outcomes.

Median (q1–q3) or Number (%)	RARC + UCS (41)	ORC + UCS (65)	LRC + UCS (22)	*p*-Value (RARC+UCS vs. ORC+ UCS)	*p*-Value (RARC+UCS vs. LRC+ UCS)
**Cystectomy surgical time, min**	140 (120–180)	110 (90–120)	105 (69–140)	**<0.0001**	**0.002**
**Diversion surgical time, min**	35 (25–45)	42 (30–50)	40 (30–50)	0.089	0.227
**Lymph node dissection surgical time, min**	40 (30–50)	52 (45–70)	60 (40–83)	**<0.0001**	**0.001**
**EBL, mL**	250 (165–410)	410 (270–620)	345 (210–605)	**0.001**	0.077
**Transfusion, n (%)**	5 (12.2%)	21 (32.3%)	5 (22.7%)	**0.019**	0.280
**Intraoperative complications, n (%)**	1 (2.4%)	6 (9.2%)	2 (9.1%)	0.170	0.236
**Type of intraoperative complications** • **Rectal injury** • **Vascular injury**	1 (2.4%) 0	4 (6.1%) 2 (3.1%)	1 (4.5%) 1 (4.5%)	0.381 0.257	0.650 0.174
**CD ≥ 4 complications, n (%)**	0	2 (3.1%)	1 (4.5%)	0.257	0.174
**LOS, days**	5 (5–9)	9 (7–10)	9 (7–10)	**<0.0001**	**0.004**
**30-day readmission, n (%)**	8 (17.7%)	13 (20%)	4 (18%)	0.770	0.976
**90-day mortality, n (%)**	3 (7.3%)	7 (10.7%)	2 (9.1%)	0.567	0.802

RARC, robot-assisted radical cystectomy; ORC, open radical cystectomy; LRC, laparoscopic radical cystectomy; UCS, ureterocutaneostomy; EBL, estimated blood loss; CD, Clavien–Dindo; LOS, length of stay. Numbers in bold indicates statistical significance.

## Data Availability

The data presented in this study are available on request from the corresponding author.

## Supplementary Materials

The following supporting information can be downloaded at: https://www.mdpi.com/article/10.3390/jcm14144898/s1, Table S1: List of abbreviations; Table S2: Fulfilled checklist of the guidelines for reporting observational studies in epidemiology (STROBE).
