# Supplementary material for: Robot-Assisted Radical Cystectomy with Ureterocutaneostomy: A Potentially Optimal Solution for Octogenarian and Frail Patients with Bladder Cancer
Source: J Clin Med. 2025 Jul 10;14(14):4898. doi: 10.3390/jcm14144898 (PMC12296046; doi:10.3390/jcm14144898)
Supplement: Supplementary file 1 [file jcm-14-04898-s001.zip › Supplementary Table S1.pdf]

Supplementary Table S1. List of abbreviation

Abbreviation	Definition
BCa	Bladder cancer
MIBC	Muscle-invasive bladder cancer
NMIBC	Non muscle-invasive bladder cancer
RC	Radical cystectomy
ORC	Open radical cystectomy
LRC	Laparoscopic radical cystectomy
RARC	Robot-assisted radical cystectomy
UCS	Ureterocutaneostomy
RIC	Italian Radical Cystectomy Registry
WHO	World Health Organization
PS	Performance Status
ASA score	America Society of Anesthesiologist score
CCI	Charlson Comorbidity Index
GFR	Glomerular filtration rate
BMI	Body mass index
EBL	Estimated blood loss
CD classification	Clavien-Dindo classification
LOS	Length of hospital stay
